# Reduced variability of neural progenitor cells and improved purity of neuronal cultures using magnetic activated cell sorting

**DOI:** 10.1371/journal.pone.0213374

**Published:** 2019-03-27

**Authors:** Kathryn R. Bowles, Julia T. C. W., Lu Qian, Benjamin M. Jadow, Alison M. Goate

**Affiliations:** 1 Department of Neuroscience & Friedman Brain Institute, Icahn School of Medicine at Mount Sinai, New York, NY, United States of America; 2 Ronald M. Loeb Center for Alzheimer’s disease, Icahn School of Medicine at Mount Sinai, New York, NY, United States of America; 3 Department of Genetics and Genomic Sciences, Icahn Institute of Genomics and Multiscale Biology, Icahn School of Medicine at Mount Sinai, New York, NY, United States of America; Lewis Katz School of Medicine at Temple University, UNITED STATES

## Abstract

Genetic and epigenetic variability between iPSC-derived neural progenitor cells (NPCs) combined with differences in investigator technique and selection protocols contributes to variability between NPC lines, which subsequently impacts the quality of differentiated neuronal cultures. We therefore sought to develop an efficient method to reduce this variability in order to improve the purity of NPC and neuronal cultures. Here, we describe a magnetic activated cell sorting (MACS) method for enriching NPC cultures for CD271-/CD133+ cells at both early (<2–3) and late (>10) passage. MACS results in a similar sorting efficiency to fluorescence activated cell sorting (FACS), while achieving an increased yield of live cells and reduced cellular stress. Furthermore, neurons derived from MACS NPCs showed greater homogeneity between cell lines compared to those derived from unsorted NPCs. We conclude that MACS is a cheap technique for incorporation into standard NPC differentiation and maintenance protocols in order to improve culture homogeneity and consistency.

## Introduction

The reprogramming of adult, fully differentiated peripheral human cells into induced pluripotent stem cells (iPSCs; [1,2]) has been a monumental breakthrough that has allowed investigators across multiple disciplines to conduct research in a diverse array of cell types from numerous individuals, resulting in the development and use of more accurate and appropriate cell culture models of human disease. Given the inaccessibility of live human brains, neuroscience in particular has benefited greatly from the ability to differentiate iPSCs into an array of neuronal and glial subtypes for the investigation and discovery of novel disease-associated phenotypes in 2D and 3D cultures [3–5]. Many protocols have been developed for the generation of iPSC-derived neural cells, most of which pass through a neural progenitor cell (NPC) intermediate before terminal differentiation into neurons or glia [6–15].

However, genetic and epigenetic variability between cell lines, as well as differences in investigator technique, cell culture conditions and differentiation protocols commonly result in imperfect NPC differentiation, which in turn impacts the purity and quality of subsequent neuronal cultures [7,9,13,15–18]. Of particular concern is the contamination of newly differentiated NPC cultures by neural crest cells (NCCs) that occurs during rosette selection; a stage of development similar to the formation of neuroepithelial cells within the neural tube [19]. NCCs, also referred to as mesenchymal stem cells, are characterized by presence of the cell surface marker, CD271 (nerve growth factor receptor; NGFR[20,21]. NCCs are multipotent cells, capable of tracing brain-associated lineages such as Schwann cells in peripheral nerves [22], as well as enteric neurons, bone and muscle cells [23], but they are not capable of differentiating into central nervous system (CNS) neurons. As such, during neuronal differentiation from NPCs, infiltrating NCCs will continue to proliferate and contaminate the neuronal culture with an undefined non-neuronal cell type. With increasing NPC passage number, NCCs will continue to proliferate and may, over time, render the NPC population unusable for neuronal differentiation. Indeed, an increased propensity for glial differentiation has been commonly reported in higher passages of NPC cultures [7,9,18]

The cell surface marker CD133 (Promonin 1) has been described as a characteristic of NPCs [17,24,25]; cells that express CD133 are positive for other common NPC markers, such as Nestin and OCT4 [24]. In addition, CD133 has proven an effective cell surface marker for isolating NPCs from human brain tissue using fluorescent activated cell sorting (FACS; [26]), suggesting that expression of this marker is a useful characteristic by which to define NPCs.

The presence of CD271+ and CD133- cells in NPC cultures has the potential to have a substantial impact on the quality and purity of neuronal cultures following differentiation; the contamination of neuronal cultures with non-neuronal, undefined cell types is problematic for the analysis of cell-autonomous neuronal phenotypes, and will add undesirable noise to data collection and analysis. Furthermore, NPC lines from different clones and individuals are likely to contain different proportions of CD271+/CD133- cells, resulting in vastly different neuronal populations and high variability in sample purity and quality. It is therefore desirable to start neuronal differentiation protocols from a uniform pool of NPCs across cell lines in order to improve the quality of NPCs, which will lead to a more homogeneous population of resulting neurons and will increase the consistency across differentiations.

To achieve this, it is essential to minimize the number of CD271+/CD133- cells in NPC cultures. One way to accomplish this is to use NPC cultures with very low (P < 3–4) passage numbers, however this is not always practical. Alternatively, FACS has been successfully used to select for CD271-/CD133+ cells, allowing for the use of NPC cultures up >50 passages [6,9,17]. The first method using early passage cultures is effective in skilled hands, although it confines the use of each cell line to a short period of time, limits the potential for expansion and requires repeated NPC differentiation from iPSCs. In contrast, while the FACS approach is highly successful in purifying NPC cultures, it is a labor-intensive, time consuming and expensive technique that results in a very low yield of live cells, as well as the potential for contamination during the process and lengthy recovery times due to slow proliferation rates [9].

Here, we describe a simple, cheap and time efficient magnetic cell sorting (MACS) method for enriching NPC cultures for CD271-/CD133+ cells, which shows a similar efficiency to FACS, while achieving a higher yield of live cells and inducing less sorting-associated cellular stress. We demonstrate that MACS results in much purer neuronal cultures following differentiation compared to unsorted cell lines, and that the protocol can be used in both early (P < 5) and late (P > 10) passage cultures for maintenance of clean NPC populations. We conclude that MACS is a cheaper, more efficient and convenient method for sorting NPCs than FACS, and that its inclusion in standard NPC-neuron differentiation protocols will greatly improve the longevity of NPC cell lines and the quality and consistency of resulting neuronal populations.

## Materials & methods

### Cell lines

Human induced pluripotent stem cells (iPSCs) were obtained from the Charles F. and Joanne Knight Alzheimer’s Disease Research Center at Washington University (S1 Table). The Icahn School of Medicine at Mount Sinai IRB reviewed the relevant operating protocols as well as this specific study and determined it was exempt from approval.

### iPSC and NPC cultures and neural differentiation

iPSCs were maintained on Matrigel (BD biosciences) in complete mTesR1 medium (StemCell Technologies) supplemented with 1% penicillin/streptomycin or 1% Antibiotic-Antimycotic (ThermoFisher Scientific) and 8μl/ml FGF2 StemBeads (StemCultures). iPSCs were passaged every ~7 days using ReLeSR dissociation reagent (StemCell Technologies) or 1mM EDTA, pH 8.0 (Invitrogen).

Neural rosettes were generated by one of two methods; F11350.1, F0510.2 and ND32915A.15 cell lines were differentiated by seeding iPSCs into v-bottomed 96 well plates in Neural Induction Media (NIM; StemCell Technologies) for 5 days in order to form 3D embryoid bodies. The resulting embryoid bodies were then transferred to Matrigel-coated plates for adherent culture and rosette formation in NIM. After 7 days, rosette selection was performed using Rosette Selection Reagent (StemCell Technologies). Selected rosettes were cultured for a further 7 days in NIM before being transferred to NPC media.

F12444-3-2, F12453-3B-1 and F13508-4-1 lines were differentiated using dual SMAD inhibition (0.1nM LDN193189 and 10μM SB431542)[27]. iPSCs maintained in Human Embryonic Stem cell (HuES) medium (DMEM/F12 (Invitrogen), 20% KO-Serum Replacement (Invitrogen), 1x Glutamax (Invitrogen), 1x NEAA (Invitrogen), 1x 2‐mercaptoethanol (Gibco)) on mouse embryo fibroblasts (mEFs) were incubated with Collagenase IV (1 mg/ml in DMEM) at 37°C for one to two hours until colonies lifted from the plate and were transferred to a non-adherent plate (Corning). Embryoid Bodies (EBs) were grown in suspension in N2/B27 media (DMEM/F12-Glutamax (Invitrogen), 1x N2(Invitrogen), 1xB27 (Invitrogen) with dual SMAD inhibitors[28,29]. After seven days, EBs were plated in N2/B27 media onto 1 mg/ml Laminin (Invitrogen)/polyornithine (PORN)-coated plates. Visible rosettes formed within one week and were enzymatically collected and plated onto matrigel-coated plates. Rosette selection was performed after 14 days of culture by Rosette Selection Reagent (StemCell Technologies).

Resulting NPCs were maintained on Matrigel-coated plates in DMEM/F12 supplemented with 1x N2, 1x B27 (both ThermoFisher Scientific), 1% Antibiotic-Antimycotic and 20ng/ml recombinant fibroblast growth factor 2 (FGF2; R&D Systems) and passaged using Accutase (Innovative Cell Technologies).

### Neuronal differentiation

Twenty-four hours after seeding on Matrigel-coated plates, NPCs were maintained in BrainPhys Neuronal medium (StemCell Technologies) supplemented with 1% Antibiotic/Antimycotic, 1x B27, 20ng/ml brain derived neurotrophic factor (BDNF), 20ng/ml glial cell line-derived neurotrophic factor (GDNF), 250ug/ml dibutyryl cyclic AMP sodium salt (cAMP) and 200μM L-Ascorbic acid (AA). Lines F12444-3-2, F12453-3B-1 and F13508-4-1 were grown in the same media supplemented additionally with 1x N2. Differentiation media was changed every 2–3 days for 4 weeks.

### Magnetic cell sorting (MACS)

All MACS antibodies, buffers and equipment were supplied by Miltenyi Biotech. Labelling and sorting protocols were carried out according to manufacturer’s recommendations using manual LD and LS selection columns, with minor adjustments. Briefly, MACS was carried out following a two-step protocol, beginning with CD271 depletion, followed by CD133 selection. We recommend a minimum input of 1 x 10^7^ NPCs for sorting, with improved cell survival and yield with an input of 2–8 x 10^7^. NPCs were therefore allowed to expand for 1–3 passages before sorting. NPCs were first dissociated by incubating with Accutase for 5 minutes at 37°C, pelleted by centrifugation at 400xg for 4 minutes, then re-suspended in MACS Separation Buffer and passed through a 40μm cell strainer to achieve a single cell suspension. Cells were incubated with Neural Crest Stem Cell Microbeads (Miltenyi Biotech, #130-097-127) for 15 minutes at 4°C, then passed through an LD separation column against a magnetic separator followed by two washes with Separation Buffer. The flow-through cells were retained as the CD271- fraction and cells retained in the column were discarded as the CD271+ NCC fraction. CD271- cells were re-pelleted and re-suspended in fresh MACS Separation Buffer, then labelled using the Indirect CD133 Microbead kit (Miltenyi Biotech, #130-091-895) for 15 minutes at 4°C. Labelled cells were then passed through an LS separation column against a magnetic separator and washed three times with Separation Buffer. Flow through cells were discarded as the CD133- fraction, and CD271-/CD133+ cells retained within the column were eluted in Separation Buffer, pelleted a final time, re-suspended in NPC media and grown in standard NPC maintenance conditions. At each stage of sorting, live cell number was determined by assessing Trypan blue negativity using a Thermo Fisher Countess Automated Cell Counter.

### Fluorescence-activated cell sorting (FACS)

Cells were dissociated by Accutase with endonuclease deoxyribonuclease I (DNase I) (Worthington Biochemical, Cat#LK003172) and passed through a 40μm cell strainer to make a single cell suspension. At least one million cells per line were conjugated with a CD271-PerCP-Cy5.5 (BD Biosciences, #560834) and CD133/1 (AC133)-PE (Miltenyi Biotech #130-080-801) antibody for 20 minutes on ice. Cells were then washed twice with chilled PBS, resuspended in 5% BSA/PBS solution and passed through a 40μm cell strainer to achieve single cell suspension. Cells were incubated with DAPI for 5 minutes and sorted on LSRII (BD biosciences) after adjustment with single color controls.

### Flow cytometry

Cells were dissociated using Accutase (Millipore), fixed for 10 min in 4% paraformaldehyde (PFA), permeabilized and blocked with 0.5% (v/v) Triton (Sigma)/1% (w/v) bovine serum albumin (BSA, Sigma) in PBS and labeled with primary antibodies S100β (mouse, 1:200; Sigma-Aldrich: S2532), GFAP (chicken, 1:200; Aves Lab), GLAST/EAAT1 (rabbit, 1:200; BOSTER: PA2185), PAX6 (rabbit, 1:200; Abcam: ab5790), and NESTIN (mouse, 1:200; Abcam: ab22035) overnight at 4°C. Following 2 washes with 1 x PBS, the cell pellet was resuspended in blocking buffer with the appropriate Alexa Fluor 488, 568, or 647 conjugated secondary antibodies (1:300, Life Technologies) for 2hr at 4°C, then washed twice more with 1 x PBS, resuspended in FACS buffer (1 x PBS (no Mg2+/Ca2+)) and filtered using a 40 μm filter (BD Biosciences). Cytometry was performed using an LSR-II (BD Biosciences) and analysis was performed using FCS Express 5 software (De Novo Software). Gating for positive cells was relative to a secondary antibody only control. Index was calculated for Marker + population as follows: % of gated cells was multiplied by geometric mean fluorescence intensity of marker + population.

### Survival assays

Immediately following either MACS or FACS, NPCs were seeded at a density of 1x 10^5^ cells per well in a 96 well plate to assay survival and stress at 24 hours after sorting. After 24 hours, media supernatant was collected for a colorimetric LDH assay (Abcam), which was carried out according to manufacturer’s guidelines and measured at 450nm with a 650nm reference on a Varioskan microplate reader. The remaining live cells were labelled with CytoCalcein violet (Abcam) and immediately imaged on a Leica DMIL LED Inverted Routine Fluorescence Microscope with a 5x and 20x objective. NPCs that had not undergone any sorting or manipulation other than regular passaging were also seeded for comparison. Additional NPCs were treated with 1μM Staurosporine (STS) for 1, 6 and 24 hours as a positive control for toxicity and cell death. The percentage of total well surface area covered by CytoCalcein violet-stained cells was determined by converting images to high contrast greyscale and submitting them for automatic thresholding and measurement in ImageJ.

### RNA extraction and qRTPCR

One million NPCs were pelleted for RNA extraction and qRTPCR analysis. For neuronal differentiation, 4x 10^5^ NPCs were seeded into one well of a 6 well plate for 4 weeks, and were then collected for RNA extraction. RNA was extracted using the RNeasy Mini kit (Qiagen) and reverse transcribed using the High Capacity RNA to cDNA kit (Thermo Fisher Scientific). For general differentiation marker analysis, qPCR was carried out using custom designed Taqman microfluidic cards (Thermo Fisher Scientific) containing 22 pluripotent, multipotent, neuronal and glial markers, as well as endogenous controls 18s ribosomal RNA and GAPDH[30]. Standard Taqman qPCR was used for the analysis of cellular stress-associated genes *CDKN1a*, *SCD*, *CCNG2 and DDIT3*, using *ActB* as endogenous control.

### Immunofluorescence

For NPC visualization, cells were seeded at 2x 10^5^ cells per well in a 24 well plate and fixed using Formalin (Sigma-Aldrich) after 24–48 hours. For neuron visualization, 1x 10^5^ NPCs were seeded for differentiation and were fixed with the same protocol after 4 weeks. All cells, other than those to be labeled with cell surface markers CD271 and CD133, were permeabilized with 0.1% Triton x-100 in PBS. All cells were blocked with 1% bovine serum albumin (BSA) in PBS. Primary antibodies against CD133 (ab19898) and Nestin (ab22035) antibodies were from Abcam and used at a dilution of 1:200 and 1:100, respectively. SOX2 (3579S; 1:400), GFAP (3670S; 1:300) and NeuN (12943; 1:500) primary antibodies were from Cell Signaling Technology. The antibody against S100β (S2532; 1:1000) was purchased from Sigma Aldrich, TUJ1 (802001; 1:500) was from BioLegend, and CD271 (MA5-13314; 1:100) was from ThermoFisher Scientific. The anti-Tau antibody (Da9; 1:200), was a kind gift from Dr. Peter Davies (Feinstein Institute for Medical Research, NY). All secondary antibodies were from ThermoFisher Scientific and used at a dilution of 1:100. Cells were imaged on a Leica DMIL LED Inverted Routine Fluorescence Microscope with a 20x objective.

### Statistical analysis

Data are represented as mean ± SEM of two to six biological replicates. Gene expression data was analyzed using the ΔΔCt method, and results were normalized to the endogenous controls. For microfluidic cards, gene expression fold change was calculated compared to *GAPDH* expression. The resulting data were subject to classical multidimensional scaling based on Euclidean distance in two dimensions, using R Studio (http://www.rstudio.com/), and principal component factor scores were compared using Tukey t-tests for uneven variance and the F-test for equal variances. Statistical significance was determined by the appropriate one-way ANOVA and Tukey post-hoc testing or using one-tailed Student’s t-test. Statistical analysis on pooled results of flow cytometry in multiple cell lines or conditions was determined by one-way ANOVA with Bonferroni correction for multiple comparisons or one-tailed Student’s t-test. All cell lines were tested in duplicate, and 5–6 cell lines were analyzed for each assay. Significant comparisons are labeled in figures as * p < 0.05, ** p < 0.01, and *** p < 0.001.

## Results

### MACS consistently isolates CD271-/CD133+ NPCs with the same efficiency as FACS while reducing cell stress and improving yield

**Fig 1:**
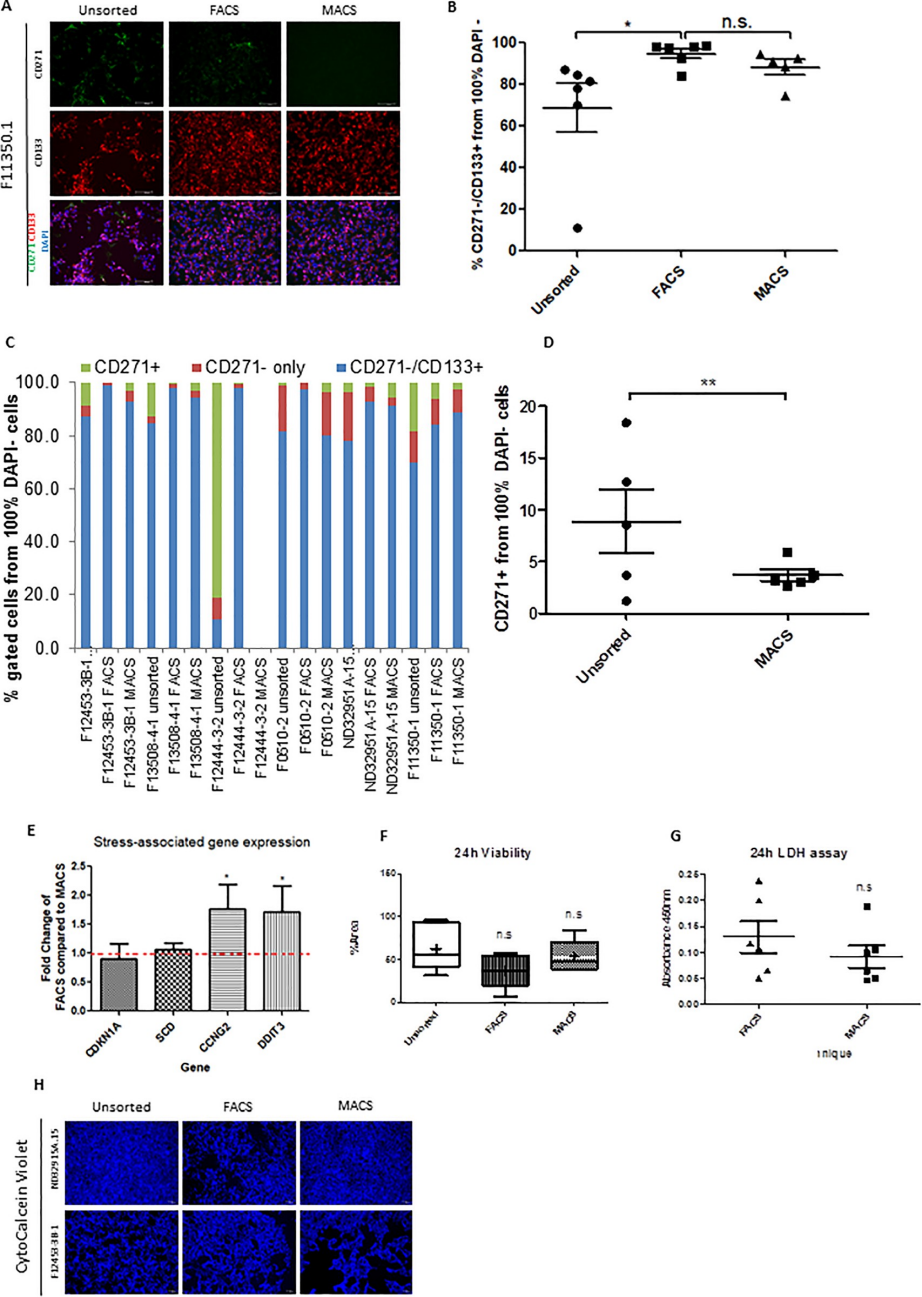
MACS reduces cell stress and improves live cell yield while maintaining an equivalent CD271-/CD133+ sorting efficiency as FACS. (A) Representative immunofluorescence images from one NPC line demonstrating a reduction in CD271+ cells and an enrichment of CD133+ positive cells following both FACS and MACS compared to unsorted cells. Cell nuclei are labelled with DAPI. Scale bar = 100μm, N = 6. (B) Combined flow cytometry analysis of percent CD271+, CD271- and CD271-/CD133+ cells from 100% live cells (DAPI negative population) comparing unsorted, FACS and MACS conditions. (C) Flow cytometry analysis in **B** presented as individual cell lines. (D) Reduction in CD271+ cell variability following MACS compared to unsorted cell**s**. **p<0.01 Variance F-test. (E) Fold change expression of stress-associated genes in cells following FACS compared to MACS. Dotted line denotes equal expression at fold change = 1. (F) Quantification of the percentage surface area covered by live cells imaged in **D**. (G) Absorbance read at 450nm of LDH assay carried out on media supernatant collected from cells 24 hours following either FACS or MACS sorting. (H) Cell viability assessed by Calcein Violet staining 24 hours following standard passage (unsorted) or following sorting by either FACS or MACS in two representative NPC lines with different survival responses. Scale bar = 100μm, *p<0.05, n.s = not significant. Error bars ± SEM.

Previous studies have found MACS to be less efficient and more variable than FACS when sorting NPCs [6,9], although these studies used different cell surface markers and sorted cells directly following rosette selection. We therefore sought to compare our MACS protocol with FACS using the same cell surface markers in NPC lines generated with two different differentiation protocols and with variable proportions of non-NPC contamination. The yield of live cells following MACS was consistently higher in all six cell lines tested, with one line showing 8 fold improvement following MACS, although this was likely a result of a mechanical error of the number of sorted cells within the flow cytometer. However, with the exclusion of this outlier, MACS still resulted in a 1.2–2.3 fold improvement (Table 1). Following MACS, we also conducted flow cytometry for CD271 and CD133 in order to directly quantify the efficiency and accuracy for isolating the desired cells. We observe a trend towards a reduced proportion of CD271+ cells following MACS (p = 0.06), and importantly the variance between lines was significantly reduced (F test for comparison of variances p = 0.0062, Fig 1D). With the exception of the failed NPC differentiation case of the F12444-3-2 line, both FACS and MACS yield 80–99% CD271 negative and CD133 positive cells from 100% DAPI negative live cells compared to 69–87% in the unsorted NPCs (Fig 1B–1D and S1 Fig, S2 Table). When comparing the CD271-/CD133+ populations of unsorted NPCs (mean = 68.65%) and FACS NPC lines (94.78%), FACS enriches the proportion of CD271-/CD133+ NPCs by 38% compared to unsorted cell lines (Fig 1B). Of note, there was no difference in the proportion of CD271-/CD133+ cells between MACS and FACS populations (mean CD271-/CD133+ cells following MACS = 88.26%) (Fig 1B), suggesting that MACS is as efficient as FACS in enrichment of CD271-/CD133+ NPCs. We confirmed successful CD271 depletion and CD133 enrichment in both FACS and MACS NPCs using immunofluorescence (Fig 1A and S1A–S1H Fig).

**Table 1:** Yield of cells following sorting with either FACS or MACS.

Cell Line	FACS input (x 10^7)	FACS output (x 10^7)	FACS Yield (%)	MACS input (x 10^7)	MACS output (x 10^7)	MACS yield (%)	MACS improvement (fold change)
F12453-3B-1	1.8	0.16	8.9	7.0	1.415	20.2	2.27
F13508-4-1	1.5	0.136	9.1	6.0	1.095	18.3	2.01
F12444-3-2	5.7	0.024	0.4	12.0	0.1	0.8	2
F0510-2	1.4	0.19	13.6	2.46	0.408	16.5	1.2
ND32951A-15	1.32	0.13	9.8	2.32	0.264	11.3	1.2
F11350-1	1.5	0.035	2.3	2.08	0.384	18.5	8

We assessed cell viability 24 hours after each sorting protocol by labelling viable cells with CytoCalcein Violet. While there was substantial variability between cell lines, there was a trend towards a reduced proportion of viable cells present 24 hours following either sorting method compared to unsorted cells, with lower viability following FACS sorting (Fig 1F–1H). It is notable that viability in a proportion of cell lines was unaffected by either sorting method, or was equivalent between MACS and FACS (S2A–S2C Fig), suggesting that MACS is at least equally if not slightly better than FACS. Analysis of LDH in the media following sorting was also highly variable between cell lines but showed a similar pattern of effect (Fig 1G); supporting the assertion that some cell lines may be intrinsically more susceptible to stress. Within individual cell lines, MACS either resulted in equivalent, or lower levels of LDH released into the media compared to FACS (S2A–S2C Fig).

Line F12444-3-2 was selected for inclusion in this study as an example of a line with very poor NPC differentiation quality; this line was unable to survive or expand following either MACS or FACS because the proportion of CD271^-^/CD133^+^ CD271-/CD133+ cells was very low (10.9% from 100% DAPI–live cells) (Fig 1B), and was therefore not included in the following experiments. In order to broadly assess whether MACS and FACS impacted other cellular stress pathways in viable cells, we looked at the expression of four stress-associated genes; Stearoyl-CoA desaturase (*SCD*; a marker for fatty-acid induced endoplasmic reticulum stress), Cyclin dependent kinase inhibitor 1A (*CDKN1A*; a marker for DNA-damage response), cyclin-G2 (*CCNG2*; a cell cycle suppressor) and DNA damage inducible transcript 3 (*DDIT3*; a marker of endoplasmic reticulum stress)[31]. While there was no significant difference between MACS and FACS cells for either *SCD* or *CDKN1A* expression, we consistently observed an increase in expression of *CCNG2* (t_18_ = 3.22, p < 0.003) and *DDIT3* (t_18_ = 1.83, p < 0.05) in multiple cell lines following FACS compared to MACS (Fig 1E and S2D Fig), suggesting increased cellular stress in FACS relative to MACS purified cells. This is consistent with our observations and other reports of slower proliferation and recovery times for NPCs following FACS [9], and supports our hypothesis that MACS is able to sort NPC cultures with the same efficiency as FACS while reducing the detrimental impact of sorting on cell stress and survival.

### CD271-/CD133+ cells are enriched for classical NPC markers SOX2 and Nestin

**Fig 2:**
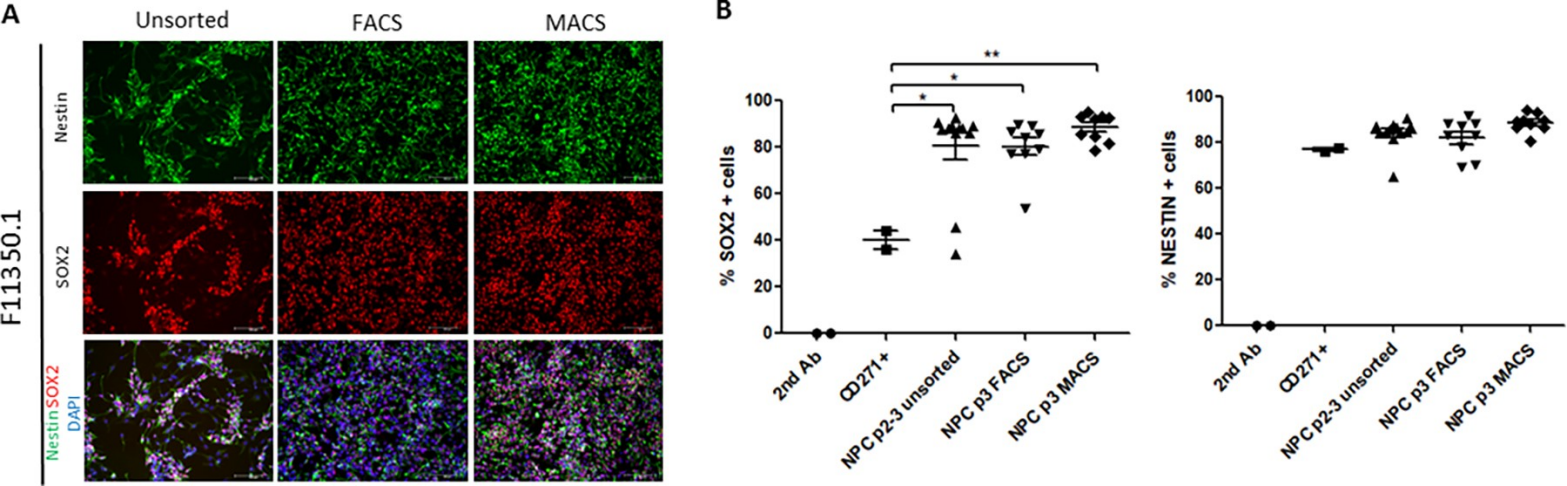
CD271-/CD133+ cells are enriched for NPC markers Nestin and SOX2. (A) Representative immunofluorescence images from one NPC line demonstrating the enrichment of Nestin and SOX2 positive cells following sorting by either FACS or MACS, compared to unsorted cells. Cell nuclei are labelled with DAPI. Scale bar = 100μm. (B). Flow cytometry analysis for SOX2 and NESTIN in unsorted cells, or following either FACS or MACS in all cell lines compared to CD271+ cells. *p<0.05, **p<0.01, Error bars ± SEM.

To confirm that isolating CD271^-^/CD133^+^ cells enriched for NPCs, we conducted immunofluorescence and flow cytometry for common NPC markers, SOX2 and Nestin, on cells prior to sorting and following either MACS or FACS protocols. In unsorted cells, we observed populations of cells that were negative for either SOX2 or Nestin staining, the proportion of which appeared to vary between cell lines (Fig 2A and S3 Fig). These are likely to be NCCs or other cell types isolated during rosette selection by STEMdiff Neural Rosette Selection Reagent and increase the variability and heterogeneity between different cell lines. Both MACS and FACS remove these cellular contaminants, leaving all remaining cells positive for SOX2 and Nestin staining. Analysis of these markers by flow cytometry confirmed that FACS and MACS both result in 90% of the cells being SOX2 and Nestin double positive (Fig 2B). Although there is cell line variability with respect to the percent of SOX2 and Nestin positive populations, FACS (t_4_ = 3.779, p < 0.05) and MACS (t_4_ = 4.568, p < 0.01) both significantly enrich for cells expressing NPC markers, particularly SOX2, compared to the CD271+ population (Fig 2B and S3 Fig). However, CD271+ cells show a similar expression of Nestin compared to CD271- cells (Fig 2B), suggesting that Nestin is not a highly specific marker for NPC populations and that SOX2 expression may be a more appropriate determinant of NPC identity in these cultures.

### MACS improves NPC culture homogeneity across cell lines and reduces undefined contaminating cell types and glial-like cells in neuronal cultures

**Fig 3:**
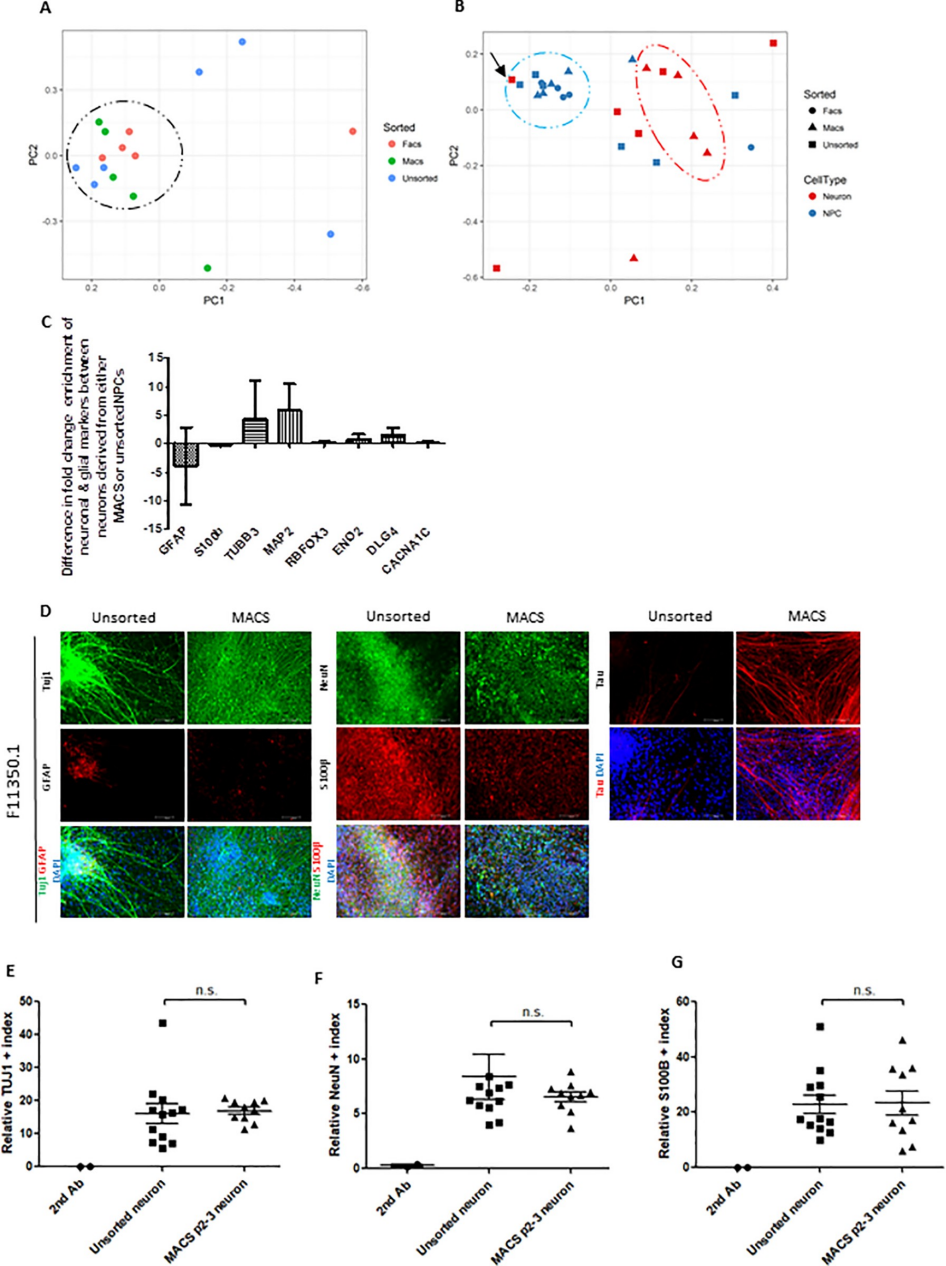
Sorting NPCs for CD271-/CD133+ cells increases homogeneity of gene expression patterns in NPCs and neurons across multiple cell lines, while enriching for neuronal-specific markers and depleting glial-specific markers in differentiated neuron cultures. (A-B). Clustering of individual cell lines using multidimensional scaling analysis based on gene expression profiles from custom designed Taqman microfluidic cards. (A)Both FACS (red) and MACS (blue) increase gene expression homogeneity between NPC lines (indicated by black circle) compared to unsorted cells (green). (B) MACS (triangles) also improves gene expression homogeneity across different neuron populations (red), as indicated by the red oval, compared to unsorted cells (squares). NPC populations are colored in blue and the homogeneous cluster is indicated by a blue circle, FACS NPCs are circles. Neurons differentiated from the unsorted F12444-3-2 NPC line that failed both FACS and MACS sorting due to low levels of CD271-/CD133+ cells are indicated with a black arrow. (C) Comparison of gene expression of glial and neuronal specific markers in neurons derived from MACS NPCs compared to neurons derived from unsorted NPCs. (D) Representative immunofluorescence images of neurons derived from NPCs demonstrating the enrichment of neuronal markers Tuj1, NeuN and Tau, as well as the depletion of glial markers GFAP and S100β when differentiated from MACS NPCs compared to unsorted NPCs. Cell nuclei are labelled with DAPI. Scale bar = 100μm, N = 5. (E-F) Flow cytometry analysis for neuronal marker TUJ1 (E) and NeuN (F) on neurons derived from MACS NPCs compared to neurons from unsorted NPCs. Relative index for each marker is generated by the multiplication of total number of fluorescent positive cells and its median fluorescence intensity. (G) Relative index of astrocyte marker, S100β by flow cytometry analysis. *p<0.05, **p<0.01, n.s = not significant. Error bars ± SEM.

The purpose of sorting NPCs before neuronal differentiation is to improve homogeneity between NPC lines, as well as between the resulting neuronal cultures following differentiation. We therefore conducted multidimensional scaling on gene expression data from 22 different pluripotency, multipotency, neuronal and glial cell markers using custom designed microfluidic cards [30] (Fig 3A and 3B). Multiple unsorted NPC cell lines failed to cluster close to the main group of NPCs, such that all unsorted cells show high variance and range in principal component 1 (PC1) between lines (variance = 2438.2, range = -84.1–40.8) across principal components one and two. After either FACS or MACS sorting these same lines now cluster with the main NPC cluster (Fig 3A). MACS sorted cells in particular demonstrate a trend towards statistically significantly reduced variance (473.3) and range (-23.7–29.5) in PC1 compared to unsorted cells (F_5,4_ = 1.55, p = 0.07), suggesting improved homogeneity of differentiation marker gene expression following sorting (Fig 3A). Following neuron differentiation, cells that have undergone MACS cluster more centrally compared to unsorted cells and have significantly reduced variance in PC1 (F_5,4_ = 10.9, p<0.05). Additionally, MACS neurons cluster significantly further away from the main NPC cluster than unsorted neurons in PC1 (unsorted neurons; t_7_ = -0.61, p = 0.28, MACS neurons; t_16_ = -3.7, p = 0.001; Fig 3B). MACS sorting therefore reduces the variability in gene expression signatures of neuronal cultures, most likely by removing unwanted cells that fail to differentiate as desired. Expression data for individual differentiation markers confirms the reduction of glial cell markers such as *S100β* (mean = -0.23 fold) and *GFAP* (mean = -3.89 fold), and an enrichment of neuronal markers *TUBB3 (4*.*37 fold)*, *MAP2 (5*.*83 fold)*, and *DLG4 (*1.61 fold) in MACS-neurons (Fig 3C), however due to the variability between NPC lines prior to sorting, these changes did not reach statistical significance.

We visualized the improvement in neuron cultures by conducting immunofluorescence for common neuronal and glial markers (Fig 3D and S4 Fig). For all cell lines, MACS drastically improved the purity of neuronal populations by removing unwanted glial cells, a significant reduction of S100β (t_2_ = 4.7, p = 0.02), while showing no difference in the TUJ1 and NeuN positive indices between unsorted neurons and MACS sorted neurons (Fig 3E and 3F and S4A–S4C Fig). We observed variability between cell lines in the proportion of glia that are generated following neuronal differentiation both before and after MACS; those cells that had undergone dual SMAD inhibition appeared to have a propensity to form S100β-expressing cells regardless of sorting, whereas those cells treated with NIM without SMAD inhibition did not; this was supported by the flow cytometry data for S100β (Fig 3G and S4D Fig). However, as the proportion of S100β-expressing cells did not appear to correlate with the extent of GFAP expression in any of the analyzed cell lines (S4 Fig) and because we did not aim to fully characterize these glial cells, it is unclear whether different NPC differentiation methods induce different states of reactivity [32] or uncharacterized glial-like cells.

### MACS efficiently isolates CD271^-^/CD133^+^ cells in late passage NPC cultures and improves neuronal purity in long-term cultures

**Fig 4:**
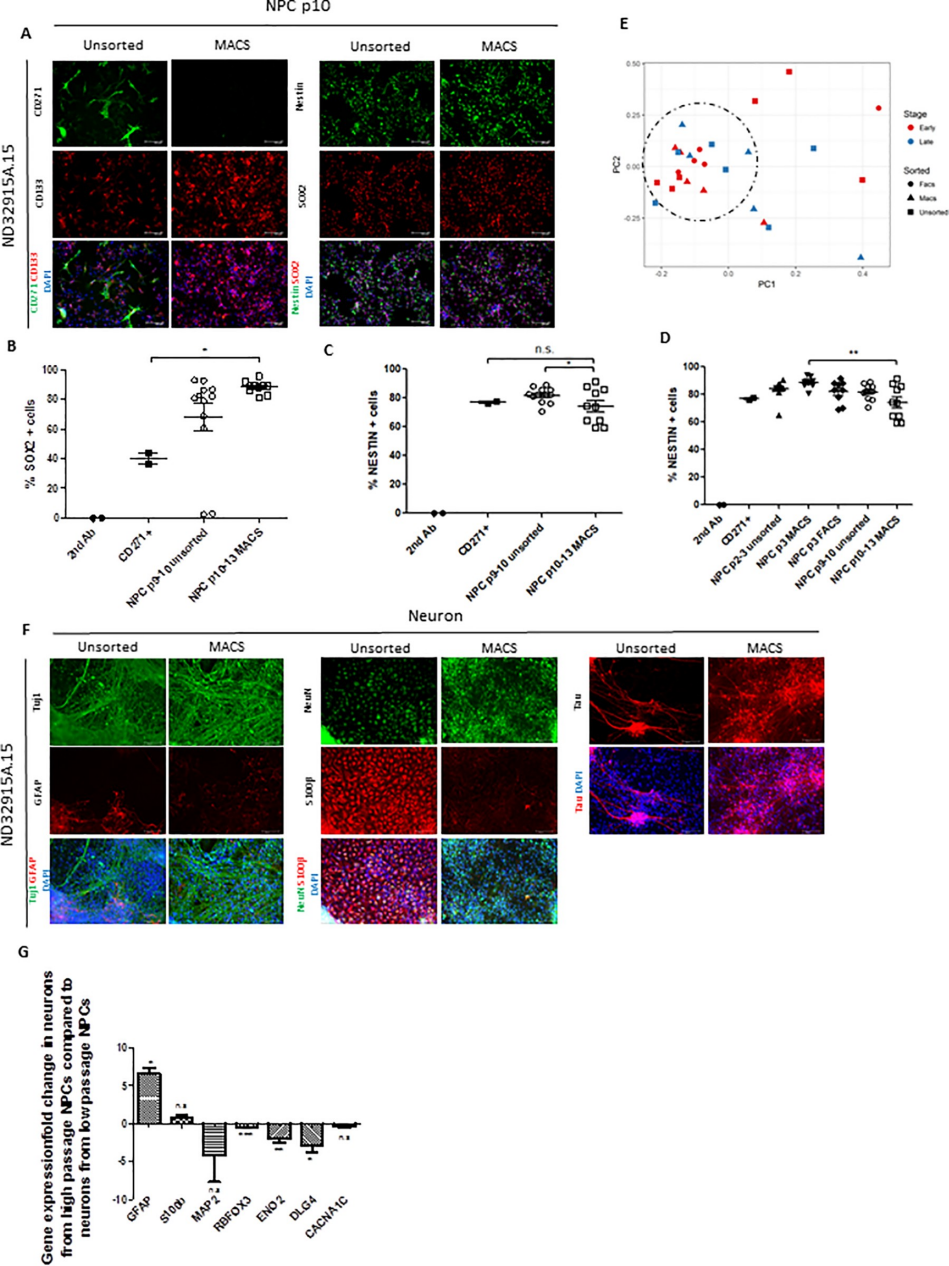
Late passage NPCs can be effectively enriched for CD271-/CD133+ cells by MACS, but still maintain a higher propensity for glial differentiation. (A) Representative immunofluorescence images from a late passage NPC line demonstrating a visible reduction in CD271+ cells and an enrichment of CD133+ positive cells, as well as an enrichment of NPC markers following MACS. Cell nuclei are labelled with DAPI. Scale bar = 100μm. (B-C) Percentage of late passage NPCs positive for SOX2 (B) and NESTIN (C) as measured by flow cytometry analysis. The summary result is shown in unsorted and MACS NPCs compared to the secondary antibody control and CD271+ cells by pooled results of individual cell lines from S5B and S5C Fig. (D) Percent NESTIN+ cells by flow cytometry analysis in early (p2-3) and late passage (p10-13) NPCs. (E) Clustering of individual late passage NPC lines using multidimensional scaling analysis based on gene expression profiles from custom designed Taqman microfluidic cards. MACS (triangles) improves homogeneity across late passage (blue) cell lines compared to unsorted cell lines (squares), and exhibits similar gene expression profiles as early passage (red) MACS and FACS (circles) NPCs, indicated by a dotted circle. (F) Representative immunofluorescence images of neurons differentiated from one late passage NPC line demonstrating an enrichment of neuronal specific markers following MACS, but a maintenance of expression of glial-specific markers. Cell nuclei are labelled with DAPI. Scale bar = 100μm. (G) Comparison of gene expression of glial and neuronal specific markers in neurons derived from late passage MACS NPCs compared to neurons derived from early passage NPCs. *p<0.05, **p<0.01, ***p<0.001. Error bars ± SEM.

It is well established that high passage (> 5) NPCs may no longer be able to effectively differentiate into neurons because of NCC or other cell type contamination and proliferation. We sought to determine whether our NPC lines with a passage number > 10 could still be cleaned using MACS, and subsequently differentiated into high quality neuron populations. As anticipated, late passage NPCs that had not been previously sorted had visible populations of CD271+ cells (Fig 4 and S5 Fig). MACS effectively removed these contaminants from NPC cultures and enriched for CD133+, Nestin and SOX2 (e.g. t_2_ = 3.58, p = 0.03) expressing cells (Fig 4A and S5 Fig). This observation was supported by the FACs data, which showed that SOX2 positive cells are significantly enriched in MACS compared to CD271+ cells (t_6_ = 8.86, p < 0.05) (Fig 4B and S3 and S5 Figs). However, Nestin expression showed line variability consistent with the previous result in earlier passage NPCs (Fig 2), but showed a significant decrease (t_20_ = 1.99, p = 0.02) in expression in late passage MACS NPCs compared to unsorted NPCs (Fig 4C; S3 and S5 Figs). Multidimensional scaling of differentiation marker gene expression clustered early and late passage NPC lines together, with an improvement in the homogeneity between lines by MACS sorting (Fig 4D).

Immunofluorescence labeling for Tuj1, NeuN and Tau in differentiated neurons again indicated a reduction in the presence of non-neuronal and unspecified cell types in neuron cultures following MACS (Fig 4E and S6 Fig). However, qPCR analysis of differentiation markers shows that late-passage NPCs have a higher propensity to form *GFAP*- and *S100β*-expressing cells following neuronal differentiation than early-passage NPCs (Fig 4F and 4G), which is consistent with existing data demonstrating that higher passage NPCs more readily differentiate into glia [7,9]. Interestingly, late passage neurons also show significantly reduced expression of typical neuron-specific differentiation markers compared to their early passage counterparts, despite undergoing the same sorting and differentiation protocol for the same length of time (*RBFOX3* t_8_ = 10.1, p <0.001, *ENO2* t_8_ = 4.4, p <0.01, *DLG4* t_8_ = 3.1, p < 0.01; Fig 4G). It is possible that this effect is an artifact due to the increased proportion of cells expressing glial markers in late-passage neuron populations, or aged NPCs may lose their propensity to develop more mature neurons within the same time frame. Regardless, MACS still greatly improved the homogeneity of neuronal cultures across the cell lines studied.

## Discussion

iPSC lines derived from multiple individuals, that have undergone independent and varied differentiation processes by diverse protocols, show substantial heterogeneity. We sought to develop a simple, effective method to reduce this variability in order to improve the purity of NPCs and their subsequent neuronal cultures. Sorting NPCs using FACS in order to increase cell line homogeneity has previously been reported and is a highly effective method of addressing this issue [6,9,17]. However, FACS can be very costly and time consuming, with a contamination risk. In addition, we and others have experienced substantial cell stress and death following FACS [9], resulting in slow proliferation and recovery time of cultures. Here, we demonstrate that MACS is an effective alternative method of enrichment for CD271^-^/CD133^+^, which is both faster and more economical than FACS.

Using MACS to refine NPC cultures has previously been reported to be extremely variable and unsuccessful [6,9], however these protocols were carried out using either a single cell-surface marker for an already differentiated neuronal cell type (PSA-NCAM;[9]) or immediately following rosette selection [6]. In contrast, we found that allowing NPCs to proliferate and expand for a few passages before undergoing a two-stage depletion and selection protocol was highly effective and consistent in multiple cell lines from two different NPC generation methods. Furthermore, we directly compared the equivalent selection procedure in the same cell lines using FACS and demonstrated that MACS results in higher yield populations of cells with the same purity as FACS processed cells. While we observed variability in the response to sorting procedures between cell lines, MACS resulted in either the equivalent or improved viability compared to FACS and consistently induced less cellular stress. Increasing yield and improving recovery time for cultures results in healthier and more robust cultures and will prevent costly delays in experimental planning and undertaking.

MACS also has the advantage of being a simple procedure that can be carried out in a cell culture hood, reducing the risk of contamination from other facilities and mechanical fault; indeed, we experienced mechanical failure when sorting one of our NPC lines using FACS, resulting in a very low yield of viable cells and weeks-long recovery of the line. These issues did not occur while using MACS. It was also far quicker to carry out MACS than FACS; MACS allows multiple cell lines to be sorted in parallel (restricted only by the number of magnets), there is no requirement for numerous controls for spectral overlap or autofluorescence that are necessary parameters to sort cells using FACS, and sorting time for MACS does not change with increased cell number.

Given its efficiency and speed, as well as being a gentler procedure with faster recovery times, we recommend MACS be used on NPC cultures as a standard step following rosette selection and prior to neuron differentiation. We have also found that most cell lines tolerate multiple MACS procedures over several weeks, and therefore it is a useful method for maintenance of high quality, high purity NPC lines. It should be noted that neither MACS nor FACS are able to rescue a low-quality cell line with a very small proportion of CD271^-^/CD133^+^ cells as shown in the F12444 line. We chose one such line for inclusion in this project to demonstrate the limitations of NPC sorting and found that while both methods were capable of isolating the desired cells, the sorting resulted in too few cells for survival or proliferation. In cases such as this when the majority of cells are CD271+ or CD133- and do not express typical NPC markers such as SOX2 or Nestin, we suggest re-differentiating from iPSC to NPC rather than trying to purify the line.

We observed the most obvious improvements in both NPC and neuron cultures following MACS by immunofluorescence, whereas the flow cytometry and gene expression data are less compelling. This is likely due to several factors; each of the NPC lines had different levels of NCC and other cell type contaminants at baseline, resulting in wide variability in initial gene expression profiles and thus also in the extent of improvement and change in NPC quality following sorting. Despite this variability, we were still able to detect trends in gene expression change in the anticipated direction following sorting, i.e. reduced glial marker expression and increased neuronal marker expression. Importantly, MACS reduced the inconsistency between NPC gene expression profiles and made them more homogeneous. It is notable that we were able to detect such differences based on a panel of only 22 genes. While many of these differences may seem insignificant, such inconsistencies between unsorted lines are likely to be far more problematic when conducting global transcriptomic profiling; using MACS will greatly reduce this risk.

Another issue relevant to both the gene expression and flow cytometry characterization data is that we do not know exactly what the infiltrating cell types are in either NPC or neuron cultures. Given the biology of rosette formation and the effectiveness of depleting CD271-expressing cells, it is probable that the majority of NPC contaminants are NCCs. However, an undefined proportion of cells are both CD271- and CD133-, and it is unclear what the differentiation potential of these cells may be and what the most relevant marker may be. Additionally, we have only assayed the non-neuronal cell types following neuron differentiation with two common glial markers, GFAP and S100β, whereas these may not be the most appropriate markers to assay the reduction of contaminants. Alternatively, the extent of undesired cell type contamination may just be too small and variable across lines to consistently and sensitively be detected by these methods.

We have demonstrated that MACS is effective in purifying both early and late passage NPC cultures, however, consistent with other observations in the literature, we observe that late passage NPCs have a greater propensity to differentiate to glia [7,9]. This effect is not likely to be associated with MACS sorting or poor differentiation of alternate cell types present in the culture, but rather a result of the inherent characteristics of NPCs [7]. It should therefore be noted that while MACS is able to reduce the presence of unwanted cell types in NPC cultures that will fail to differentiate into neurons, it is not able to prevent the inherent tendency of particular late passage NPC lines to differentiate into glia.

In conclusion, we have demonstrated that using MACS to enrich for CD271-/CD133+ cells improves the purity and quality of both NPCs and subsequent neuronal cultures. Furthermore, this technique sorts cells with the same effectiveness as FACS, but with increased yield and reduced cellular stress. Combined with its ease of use and lower cost, we propose that MACS could be incorporated into standard NPC differentiation and maintenance protocols to improve homogeneity and consistency across lines as well as over time.

## Supporting information

S1 Fig Both FACS and MACS enrich for CD271-/CD133+ cells compared to unsorted NPC lines. Related to Fig 1**. A.** Immunofluorescence for cell surface markers CD271 and CD133 from six independent NPC lines prior to and following either FACS or MACS. Line F12444-3-2 failed to survive either sorting method. **B.** Immunofluorescence for CD271 and CD133 in CD271+ cells eluted from LD columns following MACS depletion procedure, and in CD133- cells collected from LS column flow-through during the MACS selection procedure. Cell nuclei are labelled with DAPI. Scale bar = 100μm, N = 1. **C.** Flow cytometry for CD271, CD133 and DAPI. The bottom panel is for single antibody controls for CD271-PerCP-Cy5.5, CD133-PE and live cell staining for DAPI. Based on the gating, each line was sorted by CD271-/CD133+ (green population) for further analysis.
(TIF)

S2 Fig The impact of FACS and MACS on cell viability and stress is variable across cell lines. Related to Fig 1**. A.** Quantification of the percentage surface area covered by live cells as labelled with Calcein violet 24 hours following either standard passage (unsorted) or sorting by either FACS or MACS (imaged in ***B***.), or following 1–24 hours 1μM Staurosporine treatment as a positive control. N = 6, n = 2–3. *p<0.05, **p<0.01, ***p<0.001. **B.** Images of cell viability assessed by Calcein Violet staining 24 hours following standard passage (unsorted) or following sorting by either FACS or MACS in six independent NPC lines. Data is missing for F11350.1 FACS and F12444-3-2 MACS NPC lines as an insufficient number of viable cells survived sorting for analysis. Scale bar = 100μm, N = 6, n = 3. **C.** Absorbance read at 450nm of LDH assay carried out on media supernatant collected from six cell lines 24 hours following either FACS or MACS sorting, or following 1–24 hours 1μM Staurosporine treatment as a positive control. *p<0.05, **p<0.01, ***p<0.001, n = 2–3, N = 6. **D**. Fold change expression of stress-associated genes in five NPC lines following FACS compared to expression following MACS.N = 5, n = 2. N = number of cell lines, n = number of technical replicates per cell line. *p<0.05, **p<0.01, ***p<0.001. Error bars ± SEM.
(TIF)

S3 Fig Nestin and SOX2 are enriched in CD271-/CD133+ sorted NPC lines. Related to Figs 2 and 4. **A**. Immunofluorescence demonstrating the enrichment of Nestin and SOX2 positive cells in five cell lines following sorting by either FACS or MACS, compared to unsorted cells. Cell nuclei are labelled with DAPI. Scale bar = 100μm, N = 6. **B.** Flow cytometry analysis showing enrichment of SOX2 and NESTIN in MACS and FACS NPCs compared to unsorted or CD271+ cells, n = 2. *p<0.05, **p<0.01, ***p<0.001. Error bars ± SEM.
(TIF)

S4 Fig Neurons derived from MACS NPCs display an enrichment of neuronal markers and depletion of glial markers. Related to Fig 3. **A.** Immunofluorescence images of neurons derived from five NPC lines demonstrating the enrichment of neuronal markers Tuj1, NeuN and Tau, as well as the depletion of glial markers GFAP and S100β when differentiated from MACS NPCs compared to unsorted NPCs. Cell nuclei are labelled with DAPI. Scale bar = 100μm, N = 5. **B-D.** Flow cytometry analysis for neuronal markers TUJ1 and NeuN, as well as glial marker S100β on each line of differentiated neurons derived from MACS NPC compared to neurons from unsorted NPCs. *B*. Relative index for each marker is generated by the multiplication of total number of fluorescent positive cells and its median fluorescence intensity. *C*. Percent positive cells for TUJ1, NeuN and S100β in each line. *D*. Pooled results from *C*. **E**. Flow cytometry analysis gated for TUJ1, NeuN and S100β positive cells compared to each secondary antibody control on each neuron line, n = 2 technical replicates, N = 6 cell lines. *p<0.05, **p<0.01, n.s = not significant. Error bars ± SEM.
(TIF)

S5 Fig MACS enriches CD271-/CD133+ cells expressing NPC markers SOX2 and Nestin in late passage NPCs. Related to Fig 3. **A**. Immunofluorescence for cell surface markers CD271 and CD133, as well as NPC markers Nestin and SOX2 in unsorted and MACS late passage NPC lines. Scale bar = 100μm, N = 5. **B-C.** Percent SOX2+ (B) and NESTIN+ (C) cells by flow cytometry analysis of each cell line on late passage NPCs, n = 2. ***p<0.001, error bars ± SEM.
(TIF)

S6 Fig Immunofluorescence for neuronal markers TUJ1, NeuN and Tau, as well as glial markers GFAP and S100β in neurons derived from late passage unsorted and MACS NPCs. Related to Fig 4. Scale bar = 100μm.
(TIF)

S1 Table Human iPSC line clinical information. (PDF)

S2 Table % positive gated cells from 100% DAPI + cells in each condition of cell line in Fig 1C. (PDF)

## References

[1] Takahashi K, Yamanaka S. Induction of Pluripotent Stem Cells from Mouse Embryonic and Adult Fibroblast Cultures by Defined Factors. Cell. 2006;126: 663–676. 10.1016/j.cell.2006.07.024 16904174

[2] Takahashi K, Tanabe K, Ohnuki M, Narita M, Ichisaka T, Tomoda K, et al Induction of Pluripotent Stem Cells from Adult Human Fibroblasts by Defined Factors. Cell. 2007;131: 861–872. 10.1016/j.cell.2007.11.019 18035408

[3] Haggarty SJ, Silva MC, Cross A, Brandon NJ, Perlis RH. Advancing Drug Discovery for Neuropsychiatric Disorders Using Patient-Specific Stem Cell Models. Mol Cell Neurosci. 2016;73: 104–115. 10.1016/j.mcn.2016.01.011 26826498 PMC5292010

[4] Ghaffari LT, Starr A, Nelson AT, Sattler R. Representing Diversity in the Dish: Using Patient-Derived in Vitro Models to Recreate the Heterogeneity of Neurological Disease. Front Neurosci. 2018;12: 1–18. 10.3389/fnins.2018.00001 29479303 PMC5812426

[5] McKinney CE. Using induced pluripotent stem cells derived neurons to model brain diseases. Neural Regen Res. 2017;12: 1062–1067. 10.4103/1673-5374.211180 28852383 PMC5558480

[6] Cheng C, Fass DM, Folz-Donahue K, MacDonald ME, Haggarty SJ. Highly Expandable Human iPS Cell-Derived Neural Progenitor Cells (NPC) and Neurons for Central Nervous System Disease Modeling and High-Throughput Screening. Curr Protoc Hum Genet. 2018;92: 1–21. 10.1002/cphg.33 28075486 PMC5293008

[7] Paavilainen T, Pelkonen A, Mäkinen MEL, Peltola M, Huhtala H, Fayuk D, et al Effect of prolonged differentiation on functional maturation of human pluripotent stem cell-derived neuronal cultures. Stem Cell Res. Elsevier B.V; 2018;27: 151–161. 10.1016/j.scr.2018.01.018 29414606

[8] Xie Y, Schutte RJ, Ng NN, Ess KC, Schwartz PH, O’Dowd DK. Reproducible and efficient generation of functionally active neurons from human hiPSCs for preclinical disease modeling. Stem Cell Res. Elsevier B.V.; 2018;26: 84–94. 10.1016/j.scr.2017.12.003 29272856 PMC5899925

[9] Muratore CR, Srikanth P, Callahan DG, Young-Pearse TL. Comparison and optimization of hiPSC forebrain cortical differentiation protocols. PLoS One. 2014;9 10.1371/journal.pone.0105807 25165848 PMC4148335

[10] Doi D, Samata B, Katsukawa M, Kikuchi T, Morizane A, Ono Y, et al Isolation of human induced pluripotent stem cell-derived dopaminergic progenitors by cell sorting for successful transplantation. Stem Cell Reports. The Authors; 2014;2: 337–350. 10.1016/j.stemcr.2014.01.013 24672756 PMC3964289

[11] Mattis VB, Svendsen CN. Modeling Huntington׳s disease with patient-derived neurons. Brain Res. Elsevier B.V.; 2017;1656: 76–87. 10.1016/j.brainres.2015.10.001 26459990

[12] Li H, Liu H, Corrales CE, Risner JR, Forrester J, Holt JR, et al Differentiation of neurons from neural precursors generated in floating spheres from embryonic stem cells. BMC Neurosci. 2009;10: 122 10.1186/1471-2202-10-122 19778451 PMC2761926

[13] Hu B-Y, Weick JP, Yu J, Ma L-X, Zhang X-Q, Thomson JA, et al Neural differentiation of human induced pluripotent stem cells follows developmental principles but with variable potency. Proc Natl Acad Sci. 2010;107: 4335–4340. 10.1073/pnas.0910012107 20160098 PMC2840097

[14] Schwartz PH, Brick DJ, Stover AE, Loring JF, Müller FJ. Differentiation of neural lineage cells from human pluripotent stem cells. Methods. 2008;45: 142–158. 10.1016/j.ymeth.2008.03.007 18593611 PMC2528840

[15] Chambers SMSM, Fasano CACA, Papapetrou EP, Tomishima M, Sadelain M, Studer L. Highly efficient neural conversion of human ES and iPS cells by dual inhibition of SMAD signaling. Nat …. 2009;27: 275–280. 10.1038/nbt.1529.Highly PMC2756723 19252484

[16] Paşca AM, Sloan SA, Clarke LE, Tian Y, Makinson CD, Huber N, et al Functional cortical neurons and astrocytes from human pluripotent stem cells in 3D culture. Nat Methods. 2015;12: 671–678. 10.1038/nmeth.3415 26005811 PMC4489980

[17] Yuan SH, Martin J, Elia J, Flippin J, Paramban RI, Hefferan MP, et al Cell-surface marker signatures for the Isolation of neural stem cells, glia and neurons derived from human pluripotent stem cells. PLoS One. 2011;6 10.1371/journal.pone.0017540 21407814 PMC3047583

[18] Topol A, Tran NN, Brennand KJ. A Guide to Generating and Using hiPSC Derived NPCs for the Study of Neurological Diseases. J Vis Exp. 2015; 1–9. 10.3791/52495 25742222 PMC4354663

[19] Wilson PG, Stice SS. Development and differentiation of neural rosettes derived from human embryonic stem cells. Stem Cell Rev. 999 RIVERVIEW DRIVE SUITE 208, TOTOWA, NJ 07512 USA: HUMANA PRESS INC; 2006;2: 67–77. 10.1007/s12015-006-0011-1 17142889

[20] Alvarez R, Lee H-L, Hong C, Wang C-Y. Single CD271 marker isolates mesenchymal stem cells from human dental pulp. Int J Oral Sci. 2015;7: 205–212. 10.1038/ijos.2015.29 26674422 PMC5153594

[21] Álvarez-Viejo M. CD271 as a marker to identify mesenchymal stem cells from diverse sources before culture. World J Stem Cells. 2015;7: 470 10.4252/wjsc.v7.i2.470 25815130 PMC4369502

[22] Woodhoo A, Sommer L. Development of the schwann cell lineage: From the neural crest to the myelinated nerve. Glia. 2008;56: 1481–1490. 10.1002/glia.20723 18803317

[23] Huang X, Saint-Jeannet JP. Induction of the neural crest and the opportunities of life on the edge. Dev Biol. 2004;275: 1–11. 10.1016/j.ydbio.2004.07.033 15464568

[24] Liu G, Yuan X, Zeng Z, Tunici P, Ng H, Abdulkadir IR, et al Analysis of gene expression and chemoresistance of CD133+ cancer stem cells in glioblastoma. Mol Cancer. 2006;5: 1–12. 10.1186/1476-4598-5-1 17140455 PMC1697823

[25] Barraud P, Stott S, Mollgard K, Parmer M, Bjorklund A. In Vitro Characterization of a Human Neural Progenitor Cell Coexpressing SSEA4 and CD133. J Neurosci Res. 2007;85: 250–259. 10.1002/jnr.21116 17131412

[26] Uchida N, Buck DW, He D, Reitsma MJ, Masek M, Phan T V, et al Direct isolation of human central nervous system stem cells. Pnas. 2000;97: 14720–5. 10.1073/pnas.97.26.14720 11121071 PMC18985

[27] Chambers SMSM, Fasano CACA, Papapetrou EP, Tomishima M, Sadelain M Studer L. Highly efficient neural conversion of human ES and iPS cells by dual inhibition of SMAD signaling. Nat …. 2009;27: 275–280. 10.1038/nbt.1529.Highly PMC2756723 19252484

[28] Brennand K, Simone A, Jou J, Gelboin-Burkhart C, Tran N, Sangar S, et al Modeling schizophrenia using hiPSC neurons Kristen. Nature. 2011;473: 221–225. 10.1038/nature09915 21490598 PMC3392969

[29] Bardy C, Hurk M Van Den, Eames T, Marchand C, Hernandez R V, Kellogg M, et al Neuronal medium that supports basic synaptic functions and activity of human neurons in vitro. Proc Natl Acad Sci. 2015;112: E3312–E3312. 10.1073/pnas.1509741112 25870293 PMC4443325

[30] TCW J, Wang M, Pimenova AA, Bowles KR, Hartley BJ, Lacin E, et al An Efficient Platform for Astrocyte Differentiation from Human Induced Pluripotent Stem Cells. Stem Cell Reports. ElsevierCompany.; 2017;9: 600–614. 10.1016/j.stemcr.2017.06.018 28757165 PMC5550034

[31] Murray JI, Whitfield ML, Trinklein ND, Myers RM, Brown PO, Botstein D. Diverse and Specific Gene Expression Responses to Stresses in Cultured Human Cells. Mol Biol Cell. 2004;15: 2361–2374. 10.1091/mbc.E03-11-0799 15004229 PMC404029

[32] Zamanian JLJ, Xu L, Foo LCL, Nouri N, Zhou L, Giffard RG, et al Genomic Analysis of Reactive Astrogliosis. J …. 2012;32: 6391–6410. 10.1523/JNEUROSCI.6221-11.2012.Genomic PMC3480225 22553043

