# Correction: Reduced variability of neural progenitor cells and improved purity of neuronal cultures using magnetic activated cell sorting

**DOI:** 10.1371/journal.pone.0216312

**Published:** 2019-04-25

**Authors:** The PLOS ONE Staff

The co-first author’s name is spelled incorrectly. The publisher apologizes for the error. The correct name is: Julia TCW. The correct citation is:

Bowles KR, TCW J, Qian L, Jadow BM, Goate AM (2019) Reduced variability of neural progenitor cells and improved purity of neuronal cultures using magnetic activated cell sorting. PLoS ONE 14(3): e0213374. https://doi.org/10.1371/journal.pone.0213374.
