# Supplementary figures and images for: Reduced variability of neural progenitor cells and improved purity of neuronal cultures using magnetic activated cell sorting
Source: PLoS One. 2019 Mar 27;14(3):e0213374. doi: 10.1371/journal.pone.0213374 (PMC6436701; doi:10.1371/journal.pone.0213374)

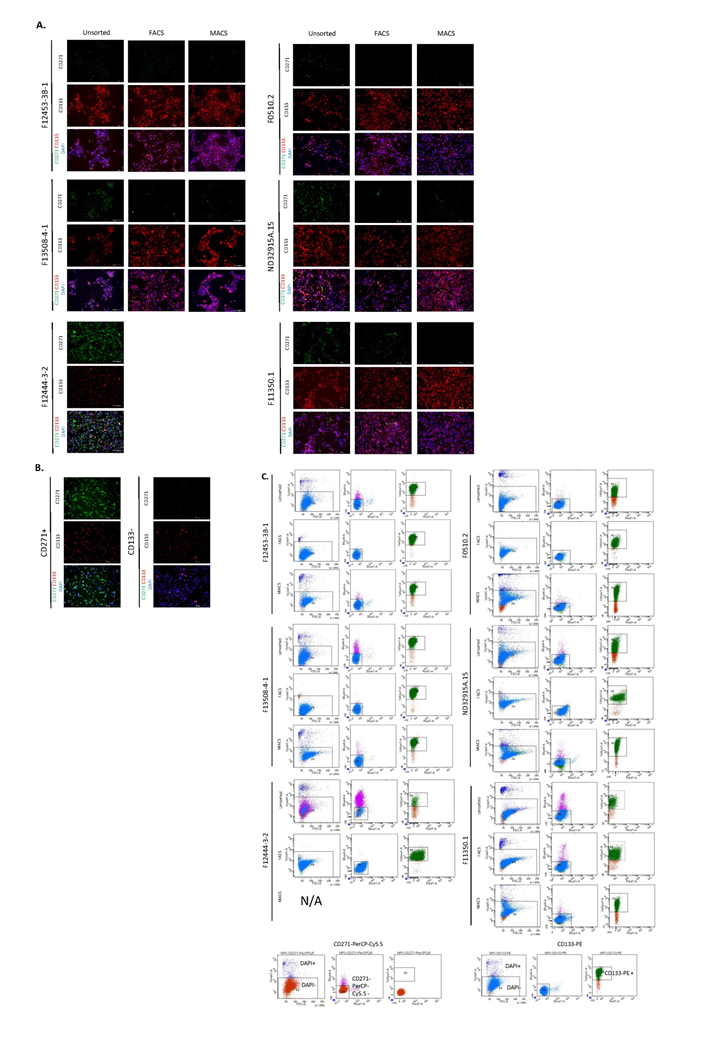

Supplement: S1 Fig — Related to Fig 1. A. Immunofluorescence for cell surface markers CD271 and CD133 from six independent NPC lines prior to and following either FACS or MACS. Line F12444-3-2 failed to survive either sorting method. B. Immunofluorescence for CD271 and CD133 in CD271+ cells eluted from LD columns following MACS depletion procedure, and in CD133- cells collected from LS column flow-through during the MACS selection procedure. Cell nuclei are labelled with DAPI. Scale bar = 100μm, N = 1. C. Flow cytometry for CD271, CD133 and DAPI. The bottom panel is for single antibody controls for CD271-PerCP-Cy5.5, CD133-PE and live cell staining for DAPI. Based on the gating, each line was sorted by CD271-/CD133+ (green population) for further analysis. (TIF) [file pone.0213374.s001.tif]

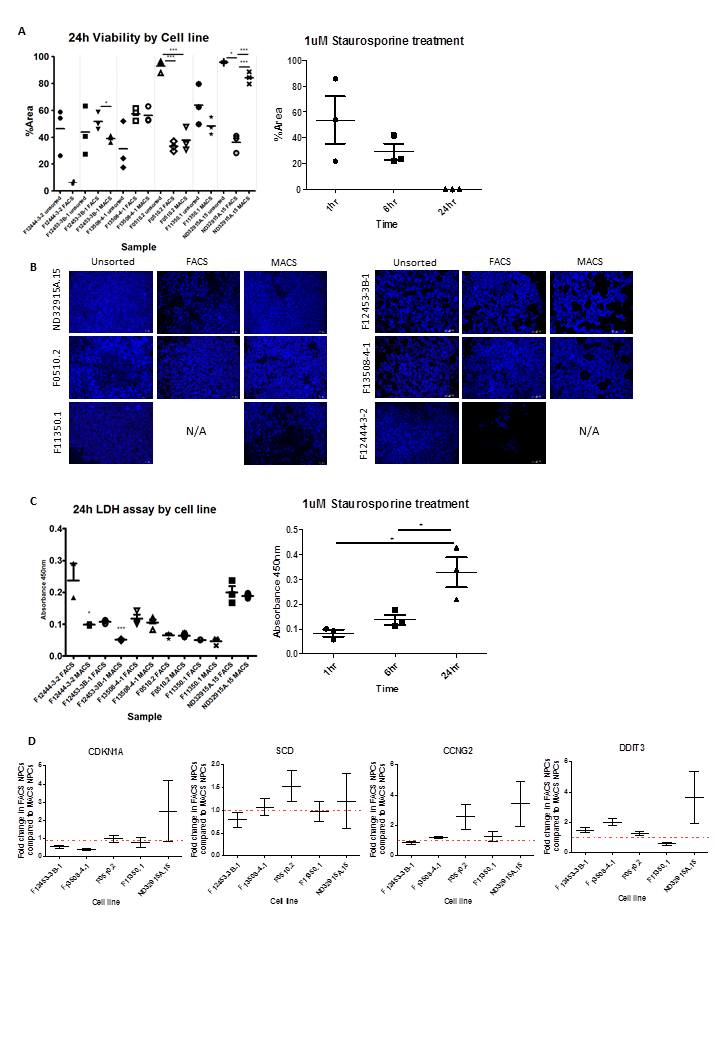

Supplement: S2 Fig — Related to Fig 1. A. Quantification of the percentage surface area covered by live cells as labelled with Calcein violet 24 hours following either standard passage (unsorted) or sorting by either FACS or MACS (imaged in B.), or following 1–24 hours 1μM Staurosporine treatment as a positive control. N = 6, n = 2–3. *p<0.05, **p<0.01, ***p<0.001. B. Images of cell viability assessed by Calcein Violet staining 24 hours following standard passage (unsorted) or following sorting by either FACS or MACS in six independent NPC lines. Data is missing for F11350.1 FACS and F12444-3-2 MACS NPC lines as an insufficient number of viable cells survived sorting for analysis. Scale bar = 100μm, N = 6, n = 3. C. Absorbance read at 450nm of LDH assay carried out on media supernatant collected from six cell lines 24 hours following either FACS or MACS sorting, or following 1–24 hours 1μM Staurosporine treatment as a positive control. *p<0.05, **p<0.01, ***p<0.001, n = 2–3, N = 6. D. Fold change expression of stress-associated genes in five NPC lines following FACS compared to expression following MACS.N = 5, n = 2. N = number of cell lines, n = number of technical replicates per cell line. *p<0.05, **p<0.01, ***p<0.001. Error bars ± SEM. (TIF) [file pone.0213374.s002.tif]

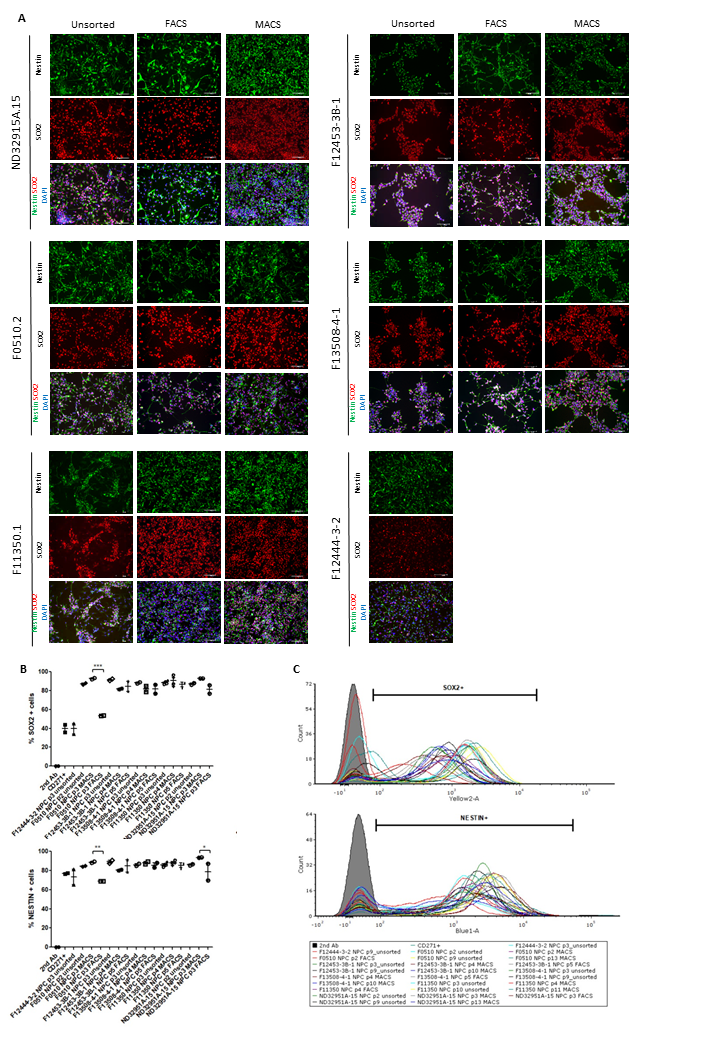

Supplement: S3 Fig — Related to Figs 2 and 4. A. Immunofluorescence demonstrating the enrichment of Nestin and SOX2 positive cells in five cell lines following sorting by either FACS or MACS, compared to unsorted cells. Cell nuclei are labelled with DAPI. Scale bar = 100μm, N = 6. B. Flow cytometry analysis showing enrichment of SOX2 and NESTIN in MACS and FACS NPCs compared to unsorted or CD271+ cells, n = 2. *p<0.05, **p<0.01, ***p<0.001. Error bars ± SEM. (TIF) [file pone.0213374.s003.tif]

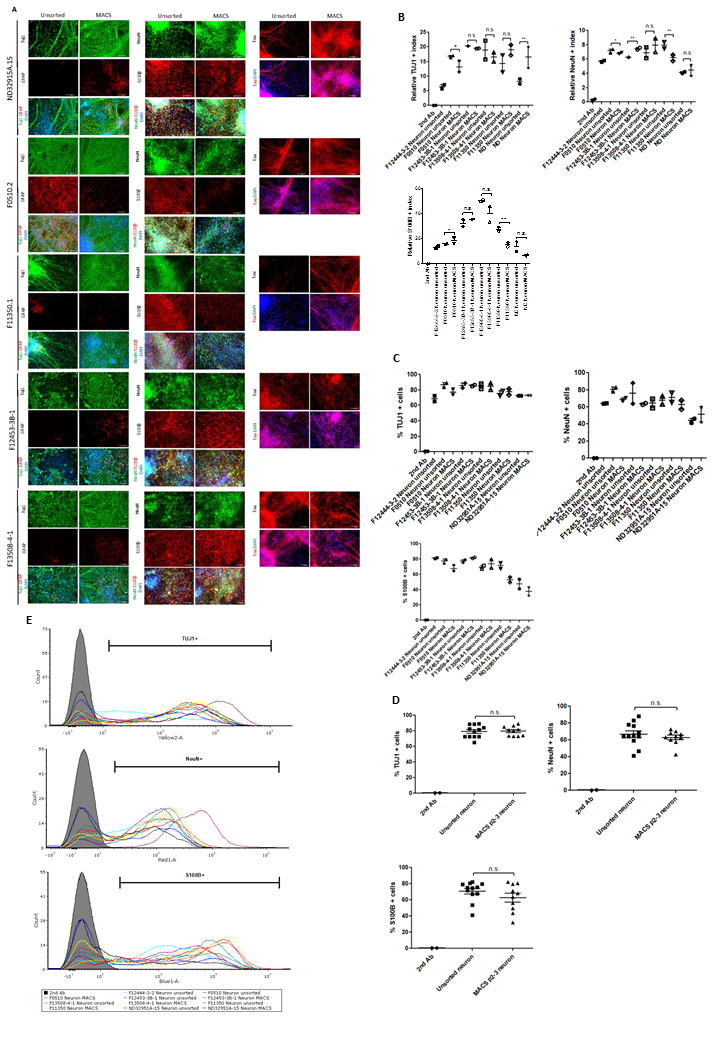

Supplement: S4 Fig — Related to Fig 3. A. Immunofluorescence images of neurons derived from five NPC lines demonstrating the enrichment of neuronal markers Tuj1, NeuN and Tau, as well as the depletion of glial markers GFAP and S100β when differentiated from MACS NPCs compared to unsorted NPCs. Cell nuclei are labelled with DAPI. Scale bar = 100μm, N = 5. B-D. Flow cytometry analysis for neuronal markers TUJ1 and NeuN, as well as glial marker S100β on each line of differentiated neurons derived from MACS NPC compared to neurons from unsorted NPCs. B. Relative index for each marker is generated by the multiplication of total number of fluorescent positive cells and its median fluorescence intensity. C. Percent positive cells for TUJ1, NeuN and S100β in each line. D. Pooled results from C. E. Flow cytometry analysis gated for TUJ1, NeuN and S100β positive cells compared to each secondary antibody control on each neuron line, n = 2 technical replicates, N = 6 cell lines. *p<0.05, **p<0.01, n.s = not significant. Error bars ± SEM. (TIF) [file pone.0213374.s004.tif]

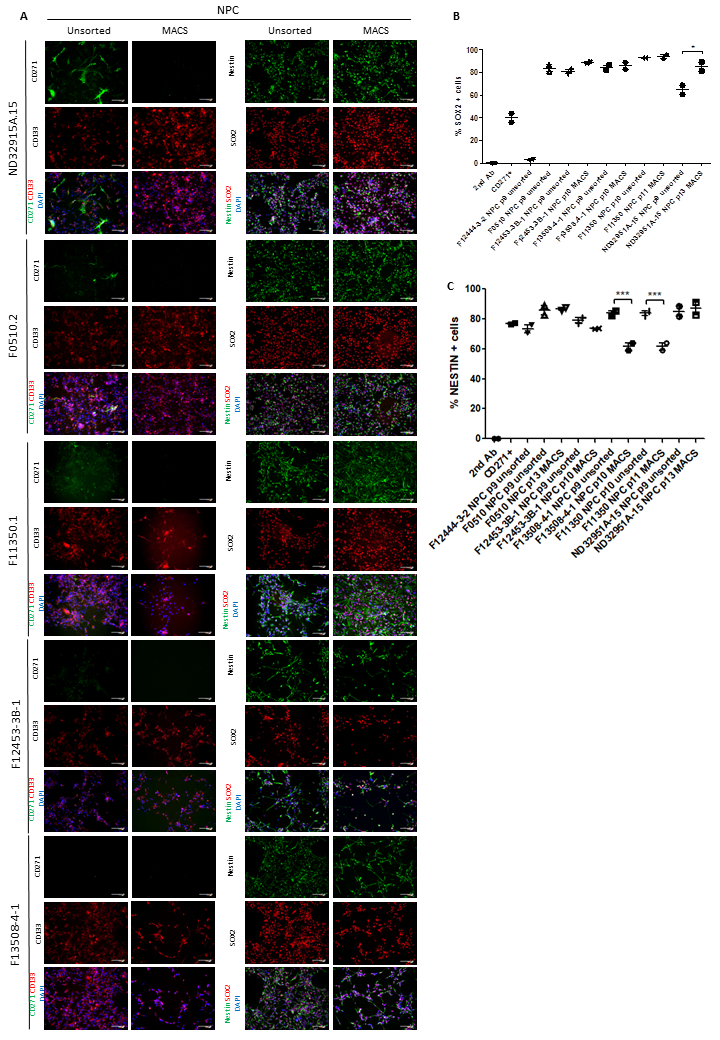

Supplement: S5 Fig — Related to Fig 3. A. Immunofluorescence for cell surface markers CD271 and CD133, as well as NPC markers Nestin and SOX2 in unsorted and MACS late passage NPC lines. Scale bar = 100μm, N = 5. B-C. Percent SOX2+ (B) and NESTIN+ (C) cells by flow cytometry analysis of each cell line on late passage NPCs, n = 2. ***p<0.001, error bars ± SEM. (TIF) [file pone.0213374.s005.tif]

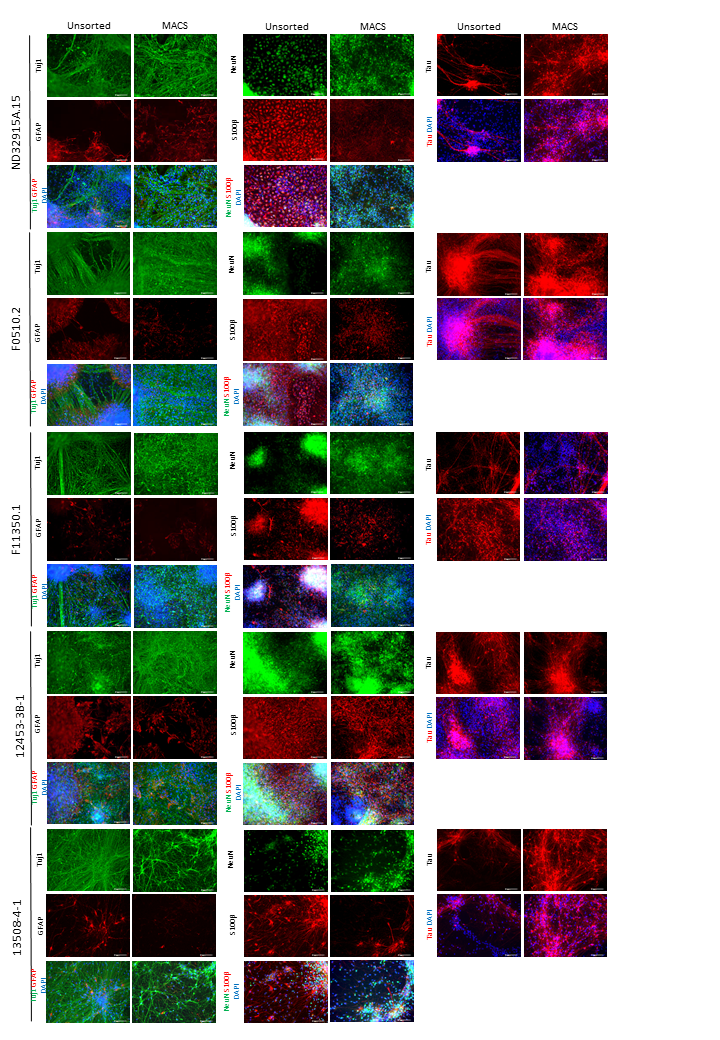

Supplement: S6 Fig — Related to Fig 4. Scale bar = 100μm. (TIF) [file pone.0213374.s006.tif]
