# Supplementary material for: Reduced variability of neural progenitor cells and improved purity of neuronal cultures using magnetic activated cell sorting
Source: PLoS One. 2019 Mar 27;14(3):e0213374. doi: 10.1371/journal.pone.0213374 (PMC6436701; doi:10.1371/journal.pone.0213374)
Supplement: S1 Table — (PDF) [file pone.0213374.s007.pdf]

**Individual ID**	**Gender**	**Age**	**Ethnicity**
F12453	Female	71	Caucasian
F13508	Female	67	Caucasian
F12444	Male	79	Caucasian
F0510	Male	Unknown	Caucasian
ND32951A	Female	Unknown	Caucasian
F11350	Male	Unknown	Caucasian
