# Supplementary material for: Reduced variability of neural progenitor cells and improved purity of neuronal cultures using magnetic activated cell sorting
Source: PLoS One. 2019 Mar 27;14(3):e0213374. doi: 10.1371/journal.pone.0213374 (PMC6436701; doi:10.1371/journal.pone.0213374)
Supplement: S2 Table — (PDF) [file pone.0213374.s008.pdf]

Cell line ID	Sorting method	CD271+	CD271-	CD271-/CD133+
F12453-3B-1	Unsorted	8.5	4.4	87.1
	FACS	0.3	1.0	98.7
	MACS	3.2	4.0	92.8
F13508-4-1	Unsorted	12.7	2.9	84.4
	FACS	0.5	1.5	98.0
	MACS	3.1	2.4	94.5
F12444-3-2	Unsorted	81.0	8.1	10.9
	FACS	0.6	1.5	97.9
	MACS	-	-	-
F0510-2	Unsorted	1.2	17.3	81.5
	FACS	0	2.6	97.4
	MACS	3.7	16.2	80.1
ND32951A-15	Unsorted	3.7	18.2	78.1
	FACS	1.5	5.8	92.7
	MACS	5.9	3.0	91.1
F11350-1	Unsorted	18.4	11.7	69.9
	FACS	6.3	9.7	84.0
	MACS	2.7	8.8	88.5
